# Metabolic and Kidney Disorders Correlate with High Atazanavir Concentrations in HIV-Infected Patients: Is It Time to Revise Atazanavir Dosages?

**DOI:** 10.1371/journal.pone.0123670

**Published:** 2015-04-15

**Authors:** Cristina Gervasoni, Paola Meraviglia, Davide Minisci, Laurenzia Ferraris, Agostino Riva, Simona Landonio, Valeria Cozzi, Nitin Charbe, Lara Molinari, Giuliano Rizzardini, Emilio Clementi, Massimo Galli, Dario Cattaneo

**Affiliations:** 1 Department of Infectious Disease, L. Sacco University Hospital, Milan, Italy; 2 Unit of Clinical Pharmacology, L. Sacco University Hospital, Milan, Italy; 3 Clinical Pharmacology Unit, Consiglio Nazionale delle Ricerche Institute of Neuroscience, Department of Biomedical and Clinical Sciences, L. Sacco University Hospital, Università degli Studi di Milano, Milan, Italy; 4 E. Medea Scientific Institute, Bosisio Parini, Italy; 5 School of Clinical Medicine, Faculty of Health Science, University of the Witwatersrand, Johannesburg, South Africa; University of Rome Tor Vergata, ITALY

## Abstract

**Introduction:**

Ritonavir-boosted atazanavir (ATV/r) is a relatively well tolerated antiretroviral drug. However, side effects including hyperbilirubinemia, dyslipidemia, nephrolithiasis and cholelithiasis have been reported in the medium and long term. Unboosted ATV may be selected for some patients because it has fewer gastrointestinal adverse effects, less hyperbilirubinemia and less impact on lipid profiles.

**Methods:**

We investigated the distribution of ATV plasma trough concentrations according to drug dosage and the potential relationship between ATV plasma trough concentrations and drug-related adverse events in a consecutive series of 240 HIV-infected patients treated with ATV/r 300/100 mg (68%) or ATV 400 mg (32%).

**Results:**

43.9% of patients treated with ATV/r 300/100 mg had ATV concentrations exceeding the upper therapeutic threshold. A significant and direct association has been observed between the severity of hyperbilirubinemia and ATV plasma trough concentrations (ATV concentrations: 271 [77–555], 548 [206–902], 793 [440–1164], 768 [494–1527] and 1491 [1122–1798] ng/mL in patients with grade 0, 1, 2, 3 and 4 hyperbilirubinemia, respectively). In an exploratory analysis we found that patients with dyslipidemia or nephrolitiasis had ATV concentrations significantly higher (582 [266–1148], and 1098 [631–1238] ng/mL, respectively) (p<0.001), as compared with patients with no ATV-related complications (218 [77–541] ng/mL).

**Conclusions:**

A significant proportion of patients treated with the conventional dosage of ATV (300/100) had plasma concentrations exceeding the upper therapeutic threshold. These patients that are at high risk to experience ATV-related complications may benefit from TDM-driven adjustments in ATV dosage with potential advantages in terms of costs and toxicity.

## Introduction

Ritonavir-boosted atazanavir (ATV/r) is one of the two protease inhibitors (PI) selected as the preferred choices in the American Department of Health and Human Services (DHHS) and European guidelines for the initial treatment of patients infected with human immunodeficiency virus-1 (HIV-1) [1,2]. This drug is relatively well tolerated in most patients; however, side effects including hyperbilirubinemia, which may result in visible jaundice or scleral icterus, dyslipidemias, nephrolithiasis and cholelithiasis have been reported in the medium and long term [3–6]. Ritonavir enhanced ATV concentrations and improved virologic activity more than unboosted ATV [7]. Nevertheless, unboosted ATV may be selected for some patients because it has fewer gastrointestinal adverse effects, less hyperbilirubinemia and less impact on lipid profiles than ATV/r.

Therapeutic drug monitoring (TDM) of ATV plasma trough concentrations is adopted for the routine management of patients only in a minority of centres worldwide. However, considerable inter-individual variability has been observed in plasma concentrations of ATV after standard dosing, mainly related to drug-to-drug interactions and inherited differences in the hepatic metabolism [8–12]. Significant correlations have been reported between plasma ATV trough concentrations and clinical outcome. In treatment-experienced as well as naïve HIV patients the highest probability of achieving undetectable viral load has been associated with ATV plasma concentrations >150 ng/mL [11–14]. Accordingly, this threshold concentration is currently recommended by international guidelines for the optimal management of patients on ATV-based antiretroviral regimens [15]. More scanty data are available concerning the relationships between ATV exposure and toxicity. A threshold ATV concentration of 800 ng/mL has been proposed as a risk factor for hyperbilirubinemia [14,16,17], whereas no specific associations have been reported for other ATV-related complications.

In the present study we sought to: I) assess the distribution of ATV plasma trough concentrations in HIV-infected patients according to drug dosage and II) verify a direct association between ATV plasma concentration and the degree of hyperbilirubinemia. As an exploratory analysis we also investigated the potential relationship between ATV concentrations and other drug-related adverse events (nephrolithiasis and dyslipidemia).

## Materials and Methods

### Study population

Male and female HIV-infected patients on ATV-based antiretroviral therapy who underwent TDM of ATV concentrations referring to the Department of Infectious Diseases at Luigi Sacco University Hospital, Milan, Italy were enrolled in the present study. Paediatric subjects, patients with severe hepatic impairment (defined as Child-Pugh Class B or C) or with history of kidney disease (including previous episodes of nephrolitiasis before initiation of ATV) were excluded from the present study. Written informed consent to patients management (that is consent for diagnostic evaluations, drug administration and all other medical procedures/interventions performed exclusively for routine treatment purposes) was collected to the first outpatient visit. Patients provided also written informed consent for their records (anonymized) to be used for future research purpose. In compliance with privacy laws, the patients’ identification code was encrypted before performing the statistical analyses. Given the retrospective observational nature of the present investigation, no formal approval from the local ethics committee was required according to the legislation of the national drug agency.

Adherence of patients to therapy was verified through direct questioning during every outpatient visits. Data on self-reporting adherence were matched with data from our Pharmacy Department in order to verify that patients have accepted the package with the antiretrovirals dose required to fully cover the time between two visits. Only patients with high adherence to antiretroviral medications (above 95% of the doses) were considered.

### Study design

This study is based on a retrospective analysis of routine TDM of ATV carried out as day-by-day clinical practice for the optimisation of drug dosing in HIV-infected patients between January 2010 and May 2013. HIV-infected patients treated with ATV for at least three months and with one assessment of ATV plasma trough concentrations were included. ATV plasma trough concentrations were stratified as below, within or above the therapeutic window, which was set at 150–800 ng/mL according to the available literature [14,16,17].

Clinical information on ATV-related complications were recorded in each patient as follows. Hyperbilirubinaemia was scored from grade 0 to grade 4 in accordance with the AIDS Clinical Trials Group Guidelines for total bilirubin levels [18]: grade 0 (<1.3 mg/dL); grade 1 (1.3–1.9 mg/dL); grade 2 (1.9–3.1 mg/dL); grade 3 (3.1–6.1 mg/dL); and grade 4 (>6.1 mg/dL). Patients with the homozygous UGT1A1*28 genotype were excluded from the analysis (higher risk to experience hyperbilirubinemia irrespective of ATV plasma concentrations). Dyslipidemia was defined by the concomitant presence of all these conditions: 1) total cholesterol >200 mg/dL or serum triglycerides >180 mg/dL; 2) at least a 20% increase in serum cholesterol or serum triglyceride levels compared to baseline; 3) normal serum lipid levels at the last visit before starting ATV. Nephrolitiasis was defined as episode of acute flank pain plus one of the following: 1) new-onset hematuria confirmed by urine analysis, 2) documented presence of stones or radiological findings suggestive of renal stones 3) stone passage confirmed by either the patient or attending physician; in the absence of urinary oxalate or urate crystals and with normal value of serum uric acid. Patients with history of nephrolitiasis before starting treatment with ATV were not included in the present study.

### Pharmacokinetic evaluations

Blood trough samples drawn into EDTA-containing vacutainers were collected from all patients immediately before the next drug intake, that is 24h or 12h after the last ATV administration in patients given the drug qd or bid, respectively (a time window of ± 30 min was directly verified by the nurse staff and considered as acceptable). All samples were centrifuged at 3000 g (+4°C), then plasma was separated and stored at -20°C. Plasma ATV concentrations were determined by a validated high-performance liquid chromatography (HPLC) method coupled with tandem mass-spectrometric detection (LC-MS/MS), based on the assay originally developed by Fayet et al [19]. Briefly, 100 microL of plasma sample was extracted by protein precipitation with acetonitrile. After centrifugation an aliquot of the supernatant was analysed by the LC-MS/MS system consisting in a Waters Alliance 2695 coupled with a Quattro Premier XE triple quadrupole (Waters, Italy). Chromatographic separation was performed under gradient conditions on a reversed phase C18 column (Xbridge, 2.1X100 mm 3.5 microm particle sizes, Waters, Milan, Italy) maintained at 50°C. Mass-spectrometric analysis was performed in positive electron spray ionisation mode. For quantification, multiple-reaction monitoring (MRM) mode was applied to monitor the transitions from the precursor ion (M+H)+ to the product ion (m/z 705.35 to m/z 335.3 for ATV). The method was linear over the ATV concentration ranges of 20 to 9000 ng/mL. The lower limit of quantification of the method set at 20 ng/mL. Between and within-day imprecision and inaccuracy were less than 15%, as requested by Consensus Guidelines on Bioanalytical Method Validations [20]. In addition our laboratory participates in an external quality control programme for the continuous monitoring of the method’s performance (the Dutch KKGT, available at http://www.kkgt.nl).

### Statistical analyses

Results were given as mean (± standard deviation) or median (plus interquartile range) according to distribution of the data based on results of the Kolmogorov-Smirnov normality test. ATV plasma trough concentrations were stratified according to drug dosage (ATV dose, frequency of daily drug administration and concomitant ritonavir use) and to therapeutic window (set at 150–800 ng/mL). All comparisons between two groups were carried out using the unpaired t test or Mann-Whitney test, whereas the ANOVA or Kruskall-Wallis tests were considered when comparing more than 2 groups, according to the distribution of the data.

The independent association between plasma ATV concentrations and dyslipidemia was assessed by means of uni- and multi-variate regression analyses using the event (toxicity yes or no) as dichotomic dependent variable and as independent covariates clinical, demographic and pharmacologic data that resulted significantly associated with the event in the univariate models (MEDCALC, Software). A p value of less than 0.05 was considered as statistically significant.

## Results

### Patients’ characteristics

Two-hundred forty HIV-infected adult patients were included in the present study. Demographic and clinical data of at the time of TDM are listed in Table 1. Patients were at 901 ± 870 days of therapy with ATV, mainly treated at the conventional ATV/r dosage 300/100 mg qd (68%), given concomitantly with tenofovir-based antiretroviral regimens (56%). Ninety-two percent of them were Caucasians. Thirty-nine (16%) out of the 240 patients evaluated (28 on ATV 300/100 mg and 11 on ATV 400 mg) had detectable viral load at the time of TDM (median, interquartile range 2.36, 1.97–3.25 log cp/mL).

**Table 1:** Main demographic, hematologic and biochemical characteristics of HIV-positive patients receiving atazanavir as part of their antiretroviral regimen.

Parameters	All Patients(n = 240)	ATV/r 300/100 (n = 163)	ATV 400 (n = 77)
Male gender, %	68%	74%	56%
Age, years	46±11	45±10	49±13
Naïve, %	23%	29%	11%
ATV therapy, days	901±870	846±886	1021±851
HAART, %	56% TDF-based	60% TDF-based	47% TDF-based
	24% ABC-based	24% ABC-based	22% ABC-based
	10% RAL-based	7% RAL-based	18% RAL-based
	10% others	9% others	13% others
Weight, Kg	69±14	70±14	68±14
Body mass index, Kg/m^2^	24±4	24±4	24±4
Creatinine, mg/dL	0.9±0.4	0.9±0.3	0.9±0.7
GGT, IU/L	72±114	85±134	47±48
ALT, IU/L	47±49	49±53	42±40
Total bilirubin, mg/dL	2.1±1.6	2.3±1.7	1.7±1.2
Total cholesterol, mg/dL	187±46	191±48	178±39
HDL cholesterol, mg/dL	50±29	50±33	49±18
Triglycerides, mg/dL	167±124	179±132	143±104
CD4, cells/mL	593±247	572±249	639±239
Pts with VL>100 cp/mL, %	12%	13%	11%
HCV/HBV coinfection, %	34%	36%	26%

TDF: tenofovir; ABC: abacavir; RAL: raltegravir; GGT: gamma-glutamyltransferase; ALT: alanine aminotransferase; HCV: hepatitis C virus; HBV: hepatitis B virus; VL: viral load

### Distribution of ATV plasma trough concentrations

A wide distribution was observed in the ATV plasma trough concentrations, with median values of 546 [204–1030] ng/mL, resulting in a mean inter-patient variability of 110.3%. Four percent of the determinations resulted <20 ng/mL. As expected, mean ATV plasma concentrations were higher in patients using ritonavir-boosted ATV than in patients using unboosted ATV (given at 400 qd or 200 bid) (650 [357–1204] vs. 238 [99–551] ng/mL, p<0.0001).

**Fig 1:**
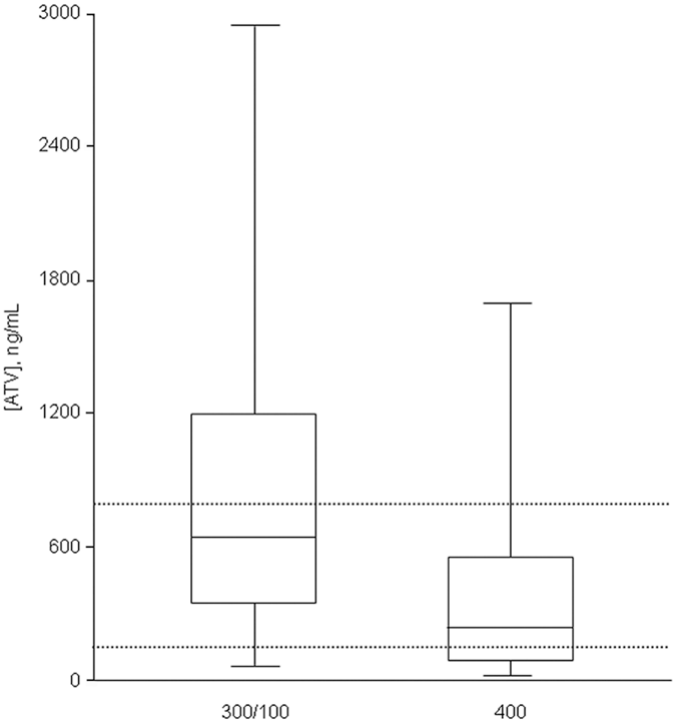
Box-plot of atazanavir (ATV) plasma trough concentrations clustered according to drug dosage. Dashed lines represent the upper and lower limits of the therapeutic window of ATV concentrations (150–800 ng/mL).

As shown in Fig 1, a significant proportion of patients with ATV plasma trough concentrations out of the therapeutic drug window were identified. 43.9% of patients treated with ATV 300/100 qd had ATV concentrations exceeding the upper therapeutic threshold, whereas unboosted ATV 400 mg was associated with the highest proportion of values <150 ng/mL (35.5%, Table 2).

**Table 2:** Distribution of atazanavir (ATV) plasma trough concentrations according to daily drug dosage.

	ATV, ng/mLmedian [IQR]	Samples <150 ng/mL	Samples150–800 ng/mL	Samples >800 ng/mL
All evaluations (n = 240)	546 [204–1030]	21.2%	44.3%	34.4%
ATV/r 300/100 mg qd (n = 164)	650 [357–1204]	11.6%	44.5%	43.9%
ATV 400 mg* (n = 76)	238 [99–551]	35.5%	44.7%	19.8%

*given either as 400 mg qd or 200 mg bid; IQR: interquartile range

### ATV plasma concentrations and hyperbilirubinemia

**Fig 2:**
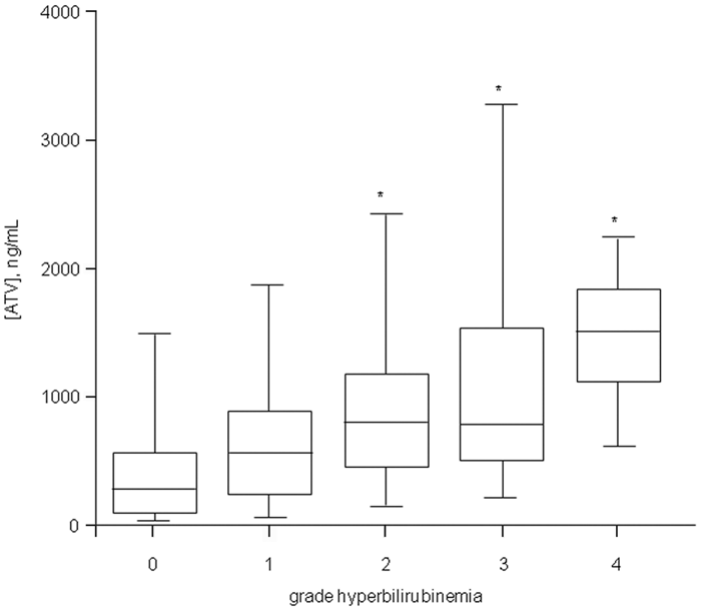
Box plot of ATV plasma trough concentrations clustered according to the grade of hyperbilirubinemia (scored as grade 0, 1, 2, 3 or 4 based on total bilirubin concentrations below 1.3 mg/dL, between 1.3–1.9, 1.9–3.1, 3.1–6.1, or above 6.1 mg/dL, respectively). *p<0.01 vs grade 0.

One-hundred forty-seven (61%) out of the 240 HIV-positive patients enrolled in the present study experienced hyperbilirubinemia of grade ≥1. As shown in Fig 2, a significant and direct association has been observed between the severity of hyperbilirubinemia and ATV plasma trough concentrations (ATV concentrations: 271 [77–555], 548 [206–902], 793 [440–1164], 768 [494–1527] and 1491 [1122–1798] ng/mL in patients with grade 0, 1, 2, 3 and 4 hyperbilirubinemia, respectively, with p<0.01 of grade 2, 3, and 4 vs. grade 0). The same trend was confirmed also when repeating the above mentioned comparisons stratifying data according to boosted or unboosted ATV intakers (Table 3).

**Table 3:** Atazanavir (ATV) plasma trough concentrations measured in patients that did or did not experienced drug-related adverse events.

Adverse event	n	Overall patients	n	ATV/r 300/100	n	ATV 400
Hyperbilirubinemia						
- Grade 0	86	271 (77–555)	52	347 (121–785)	34	141 (56–293)
- Grade 1	45	548 (206–902)	30	634 (355–962)	15	194 (106–358)
- Grade 2	54	793 (440–1164)*	40	909 (586–1264)**	14	466 (260–804)**
- Grade 3	41	768 (494–1527)*	31	825 (531–1368)**	10	540 (394–1979)*
- Grade 4	7	1491 (1122–1798)*	6	1363 (1066–1693)**	1	1836
Dyslipidemia	55	582 (266–1148)*	44	746 (305–1313)**	11	250 (106–486)
Nephrolitiasis	11	1098 (631–1238)*	9	1098 (652–1285)**	2	742 (293–1191)
Controls^	66	218 (77–541)	39	343 (125–810)	27	141 (50–259)

Data were stratified also according to ritonavir use.

^patients that did not develop ATV-related adverse events;

*p<0.01 and

**p<0.05 vs. controls

### ATV plasma concentrations and other drug-related adverse events

**Fig 3:**
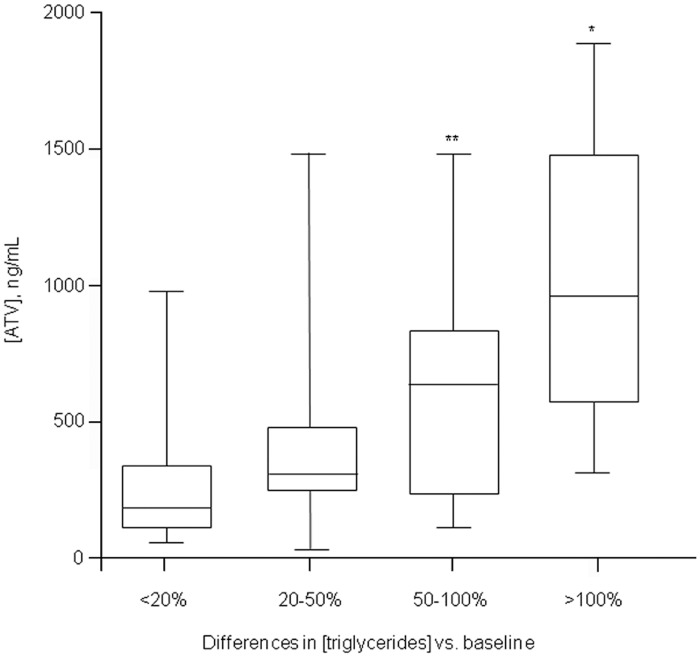
Box plot of ATV plasma trough concentrations clustered according to the magnitude of triglyceride concentrations increase as compared to values measured at the last visit before starting ATV. *p<0.01 and **p<0.05 vs. patients experiencing ≤20% increase (first column from the left).

Overall, 55 out of the 240 HIV-positive patients developed dyslipidemia, namely hypercholesterolemia (n = 26, mean increase 46±22%) or hypertriglyceridemia (n = 45, mean increase 114±76%). Patients with dyslipidemia had ATV concentrations significantly higher as compared with patients with no ATV-related complications (582 [266–1148] vs. 218 [77–541] ng/mL, p<0.01). This was confirmed also when repeating the above mentioned comparisons according to ritonavir or not administration (Table 3). Moreover, in order to demonstrate a direct relationship between ATV concentrations and lipid increase, we attempted to correlate ATV concentrations with the magnitude of lipid levels increase as compared to baseline. Using this approach we found a direct association between ATV concentrations and the percentage increase in serum triglycerides levels (Fig 3). A similar, but not significant trend, was also observed for cholesterol (data not shown).

At univariate analysis ATV concentrations (p<0.001), concomitant HAART (p = 0.079), ritonavir use (p = 0.009) and patients’ gender (p = 0.046) were associated with the development of dyslipidemia, while no association was found with other clinical covariates (Table 4). In the multivariable regression analysis only ATV concentrations (p<0.01) and ritonavir use (p = 0.02) remained independently associated with dyslipidemia.

**Table 4:** Univariate and multivariate regression analysis of clinical, demographic and pharmacological covariates associated with the development of atazanavir (ATV)-related dyslipidemia.

	Univariate		Multivariate	
Variable	r-value	p-value	r- value	p-value
Gender	0.14	0.046		
Age	0.04	0.502		
Body weight	0.04	0.469		
Serum creatinine	0.01	0.616		
ALT	<0.01	0.276		
GGT	0.01	0.172		
HCV/HBV Coinfection	0.01	0.873		
CD4 count	0.02	0.336		
Concomitant ARV drugs	0.12	0.079		
Days of ATV therapy	<0.01	0.980		
ATV concentrations	0.35	<0.001	0.33	<0.01
Ritonavir use	0.18	0.009	0.18	0.02

r-value: correlation coefficient; GGT: gamma-glutamyltransferase; ALT: alanine aminotransferase; HCV: hepatitis C virus; HBV: hepatitis B virus; ATV: atazanavir

Eleven out of the 240 HIV-positive patients enrolled in the present study experienced episodes of nephrolitiasis indirectly attributed to ATV after having excluded other clinical conditions predisposing to kidney stones. As shown in Table 3 these patients were mainly treated with ritonavir (82%) and had ATV concentrations significantly higher as compared with patients with no ATV-related complications (1098 [631–1238 vs. 218 [77–541] ng/mL, p<0.01). Only one out of 240 patients experienced an episode of cholelithiasis 226 days after starting treatment with ATV (ATV plasma trough concentrations: 438 ng/mL).

## Discussion and Conclusion

In this study, we document that a significant proportion of HIV-infected patients treated with ATV at the standard 300/100 mg qd dosage had plasma drug concentrations largely exceeding the upper therapeutic threshold, whereas nearly one third of patients treated with unboosted ATV had drug concentrations below 150 ng/mL, considered as the minimum concentration to be reached for the optimal management of patients on ATV-based antiretroviral regimens [15].

More than 60% of HIV-positive patients enrolled in the present study experienced hyperbilirubinemia of grade 1 or more, allowing us to deeply investigate the potential contribution of ATV concentrations on this drug-related adverse event. In our study we firstly confirmed the previously reported relationship between ATV exposure and the increase in total bilirubin concentrations [14,16,17]. Moreover, we extended previous findings by documenting a direct and linear correlation between ATV trough concentrations and the severity of hyperbilirubinemia. Interestingly, such relationship was established also when excluding patients homozygous for the UGT1A1*28, the genotype associated with a defect in the metabolism of bilirubin [21,22]. Using this approach, we demonstrated that association of ATV exposure with hyperbilirubinemia still persisted also in patients with the favourable UGT genotype. Accordingly, it could be speculated that in these patients, lacking of an inherited risk to experience the event, ATV-related hyperbilirubinemia might be eventually reversed by reducing ATV doses.

The potential effect of ATV on lipid profile is still a matter of debate. Meta-analyses have reported that plasma lipid concentrations were lower with ATV/r than with other ritonavir-boosted PI regimens [23]. Nevertheless, evidence is also available showing that ATV has a worst atherogenic lipid profile compared with non nucleoside reverse transcriptase inhibitors [6]. Our exploratory analyses study extended previous findings by documenting that also dyslipidemia might be associated with ATV plasma trough concentrations. In order to demonstrate a direct relationship between ATV exposure and lipid increase, we correlated ATV concentrations with the magnitude of lipid levels increase as compared to baseline. Using this approach we found a linear and significant association between ATV concentrations and the percentage increase in serum triglycerides levels, but not for cholesterol, probably because of the less number of events and the narrow distribution in the percentage increased compared with triglyceride concentrations. Among the different ATV-based regimens, lipid alterations were more frequently found in patients on ATV/r that in those given ATV alone [7], suggesting a key role of ritonavir [24]. This has been fairly confirmed also by our study by documenting that: I) patients experiencing ATV-related complications were more frequently receiving ritonavir; II) the significant association between ritonavir and ATV-related dyslipidemia was confirmed by multivariate analyses. In order to avoid the potential bias related to ritonavir use associated to the fact that a higher proportion of patients in the “adverse effect group” were treated with boosted ATV compared with control patients (those who did not experienced ATV-related complications), we repeated our comparisons stratifying data according to ritonavir boosted/unboosted intakers. Also using this approach, we documented that among patients given ATV/r 300/100, those experiencing dyslipidemia have significantly higher ATV concentrations compared with those not experiencing drug-related complications. A similar, but not significant, trend was found also in patients treated with ATV 400 mg. The small number of ATV unboosted intakers did not allow us to reach definitive conclusion on this topic. We are, however, confident that our preliminary findings could provide the rationale for ad-hoc prospective studies aimed at investigating the impact of ATV exposure on dyslipidemia in unboosted regimens. We also acknowledged that, as an important additional limitation, no information were available on ritonavir exposure, because ritonavir concentrations are not assessed in the routine management of HIV patients. Accordingly, the potential contribution of ritonavir co-administration on episodes of dyslipidemia in patients treated with ATV cannot be completely ruled out.

Cases of complicated and uncomplicated ATV-associated nephrolithiasis have been described, sometimes leading to obstructive uropathy and acute renal failure [25–28]. The association between exposure to ATV and increased incidence kidney stones has been subsequently confirmed also by epidemiological studies [5,29]. To the best of our knowledge, our is the first report showing that overexposure to ATV is associated also with increased risk of nephrolitiasis. It should be, however, acknowledged that as potential limitation of the present observation, the presence of ATV in the stones was not directly verified, and that episodes of drug-related nephrolitiasis were diagnosed indirectly after excluding the presence of oxalate or urate crystals in urinalysis and high value of serum uric acid.

The potential association between exposure to ATV and increased risk to cholelithiasis is still a matter of debate. Indeed, Hamada and co-workers reported a low incidence of complicated cholelitiasis in patients on ATV comparable to other PIs [30]. These results have been partially challenged by recent findings from the same authors [31] reporting an increased risk of cholelithiasis in patients treated with ATV for more than two years. In our cohort, we found only one episode of cholelithiasis in a patient treated with ATV for less than one year.

In conclusion, in this study we documented that significant proportion of patients treated with conventional ATV dosages had plasma concentrations exceeding the upper therapeutic threshold. A likely possibility is an inherited deficit in ATV clearance [32] and/or ATV metabolism; in particular, ATV is a dedicated CYP3A substrate, which includes 3A4 and 3A5, two polymorphic genes [8,9,10]. The administration of unboosted ATV to healthy subjects carrying the defective CYP3A5*3 resulted in significantly higher ATV concentrations compared with values measured in patients expressing CYP3A5 [21]. Assuming that over 90% of patients in our study were Caucasians with a high prevalence of carriers of CYP3A5 *3, it is likely that the observed overexposure to ATV concentrations is the result of excessively high dose of ritonavir-boosted ATV or of needless boosting with ritonavir. This is a first important conclusion of our study that raises concerns on the need of full dose of ritonavir-boosted ATV in Caucasian patients and opens new questions about the ATV dosages that should considered correct (or in label and off label in Europe). This may be particularly relevant in light of the recent findings showing that ritonavir may accumulate at intracellular level at higher degree than other PIs providing additional antiviral activity and eventually allowing the use of reduced PI doses [33]. We also found that such patients have the highest risk of experiencing ATV-related complications and may benefit from TDM-driven adjustments in ATV dosage with potential advantages in terms of costs and toxicity.
